# The impact of the COVID-19 pandemic on children and adolescent mental health inpatient service use in England: An interrupted time-series analysis of national patient records

**DOI:** 10.1192/j.eurpsy.2023.743

**Published:** 2023-07-19

**Authors:** A. Tsiachristas, J. Holland, B. Guo, K. Sayal, A. Pari

**Affiliations:** 1 Department of Population Health, University of Oxford, Oxford; 2 University of Nottingham, Nottingham; 3 NHS England, London, United Kingdom

## Abstract

**Introduction:**

During the COVID 19 pandemic children and young people (CYP) faced significant restrictions. The virus and restrictions also affected how health services could function, including mental health. Research analysing the COVID 19 pandemic is important to ensure dynamic and resilient service design in case of future national emergencies.

**Objectives:**

To investigate the impact of COVID-19 lockdowns on CYP psychiatric admission trends during lockdowns 1 (started 26 March 2020) and 2 (started 20 November 2020) of the COVID 19 pandemic in England.

**Methods:**

Routinely collected, retrospective, English, administrative data looking at the CYP hospital admissions, length of stay and patient demographics were analysed using an interrupted time series analysis to compare pre-pandemic service use with service use during COVID 19 lockdowns 1 and 2. The analysis used an ordinary least squares (OLS) approach with Newey–West standard errors to handle autocorrelation and heteroscedasticity.

**Results:**

**Table 1.** Patient characteristics in the entire sample (n=6,250)

**Variable**	**Pre-pandemic**	**Post-pandemic**	**Total sample**
	**(n=1,156)**	**(n=94)**	**(n=6,250)**
Mean age at admission (SD) [Median; IQR]	15.3 (1.7) [16;3]	15.6 (1.6) [16;2]	15.3 (1.7) [16;3]
Gender			
Female	70%	72%	70%
Missing	1%	2%	1%
BAME background			
Yes	18%	18%	18%
Missing	7%	6%	7%
Looked after			
Yes	11%	8%	11%
Missing	14%	13%	14%
In full education			
Yes	43%	34%	43%
Missing	35%	47%	35%
Mean number of admissions per patient (SD) [Median; IQR]	1.7 (1.2) [1;1]	1.2 (0.6) [1;0]	1.7 (1.2) [1;1]
Mean length of stay (SD) [Median; IQR] n	93 (94) [68;94] 6,065	65 (65) [43;77] 71	93 (94) [67;94] 6,136
Mean number of out-of-area admissions per patient (SD) [Median; IQR]	0.48 (0.8) [0;1]	0.33 (0.6) [0;1]	0.48 (0.80) [0;1]

SD: standard deviation; IQR: Interquartile range

**Image:**

**Figure:**
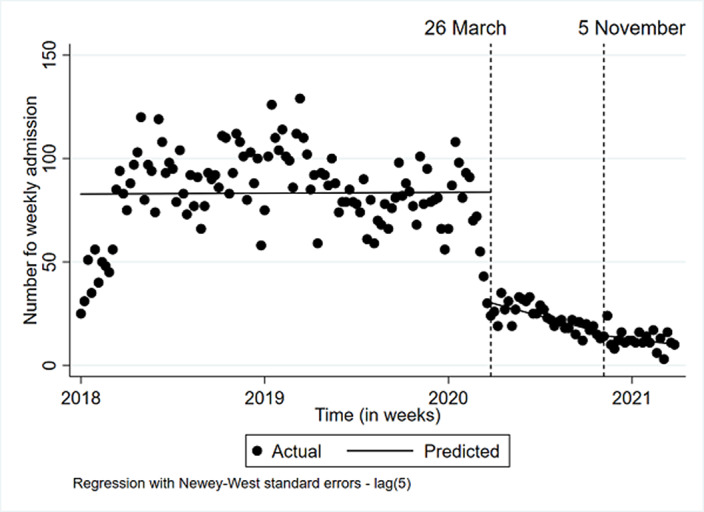

**Image 2:**

**Figure:**
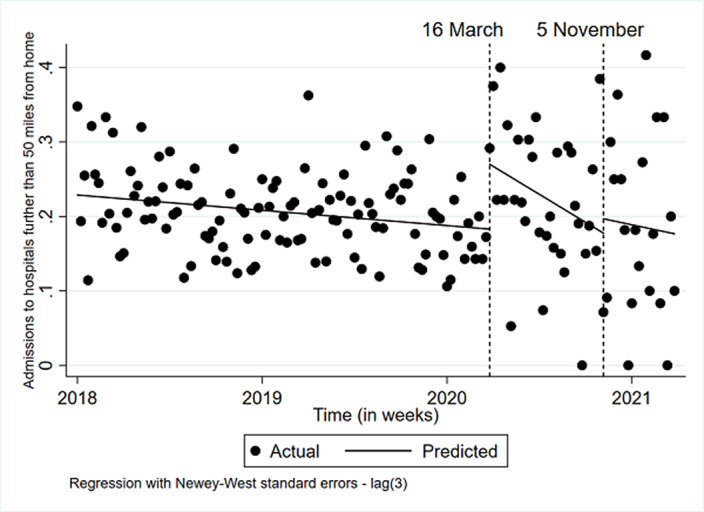

**Image 3:**

**Figure:**
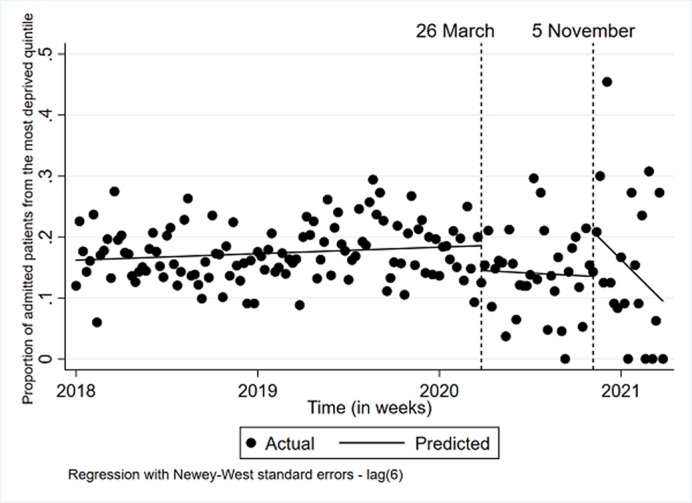

**Conclusions:**

During lockdown 1 and 2, psychiatric admissions for CYP were fewer and shorter. The rise in admissions for more deprived CYP and looked after children suggests these CYP may have been disproportionately affected by the pandemic.

**Disclosure of Interest:**

None Declared

